# Le Syndrome Kid ou Keratitis-Ichthyosis-Deafness Syndrome: à propos d'un cas

**DOI:** 10.11604/pamj.2014.19.233.5215

**Published:** 2014-10-31

**Authors:** Zineb Khrifi, Hicham Tahri

**Affiliations:** 1 Service d'Ophtalmologie, CHU Hassan II de Fès, Maroc

**Keywords:** Syndrome Kid, kératite, ichtyose folliculaire, Kid Syndrome, keratitis, follicular ichthyosis

## Image en medicine

Le syndrome KID est une anomalie congénitale rare des tissus d'origine ectodermique, caractérisé par une kératite bilatérale progressive accompagnée de néovaisseaux (keratitis), une atteinte cutanée érythro-kératodermique et/ou ichtyosique (ichtyosis) et une surdité de perception sévère (deafness). Nous rapportons le cas d'un jeune garçon de 12 ans, suivi depuis deux ans pour ichtyose, qui consulte pour une photophobie intense et une baisse de l'acuité visuelle. Il ne présente aucun antécédent familial identique. L'interrogatoire de la mère révèle une surdité depuis la petite enfance. L'examen ophtalmologique est difficile vu la photophobie et l'acuité visuelle reste difficile à chiffrée à cause de la surdité, elle est estimée au décompte des doigts à 4 mètres au niveau des deux yeux. L'examen à la lampe à fente trouve un épaississement et une kératinisation des bords palpébraux, une kératite ponctuée superficielle diffuse, un syndrome sec sévère et une importante néo vascularisation stromale superficielle bilatérale. L'examen général trouve une alopécie importante, une perte des sourcils, une érythrodermie, un léger retard staturo-pondéral, des fissures cutanées et une infection secondaire à Trichophyton rubrum du cuir chevelu. L'audiogramme montre une surdité de perception bilatérale, et la biopsie cutanée objective une kératose lamellaire compatible avec une ichtyose folliculaire. L'enfant est mis sous un traitement à base de larmes artificielles en instillation horaire avec une hygiène des paupières. Les lésions cutanées sont traitées symptomatiquement par de la vaseline, avec un traitement antimycosique de sa teigne.

**Figure 1:**
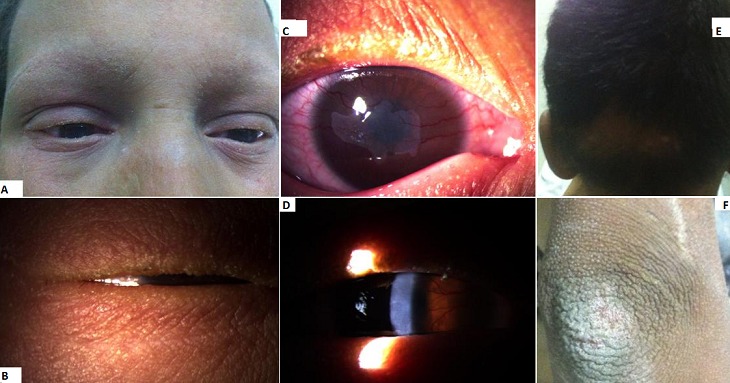
(A) image montrant une perte des sourcils, une érythrodermie et quelques fissures cutanées; (B) perte des cils et kératinisation des bords palpébraux; (C) opacités cornéennes avec une néovascularisation périphérique circonférentielle progressant vers le centre; (D) opacités et néovascularisation cornéennes; (E) infection secondaire à Trichophyton rubrum du cuir chevelu; (F) peau sèche, rugueuse avec hyperkératose au niveau du coude

